# Foreign Body Ingestion in Pediatrics: Distribution, Management and Complications

**DOI:** 10.3390/medicina55100686

**Published:** 2019-10-14

**Authors:** Jiraporn Khorana, Yaowaret Tantivit, Chanitsara Phiuphong, Saranchana Pattapong, Suparat Siripan

**Affiliations:** 1 Division of Pediatric Surgery, Department of Surgery, Faculty of Medicine, Chiang Mai University Hospital, 110 Intavaroros Road, Muang Chiang Mai District, Chiang Mai 50200, Thailand; 2 Clinical Epidemiology and Statistical Statistic Center, Faculty of Medicine, Chiang Mai University, Chiang Mai 50200, Thailand; 3 Faculty of Medicine, Chiang Mai University Hospital, Chiang Mai 50200, Thailand; ayeyaowaret@gmail.com (Y.T.); am.chanissara@gmail.com (C.P.); eve.plathong@gmail.com (S.P.); suparatsiripan@gmail.com (S.S.)

**Keywords:** foreign body ingestion, pediatric, endoscopy, guidelines

## Abstract

*Background and Objectives:* Foreign body (FB) ingestion is a common problem in children, causing serious complications. This study aimed to identify the distribution of types and locations of these foreign bodies and create Chiang Mai University (CMU) Guidelines. *Materials and Methods:* A retrospective descriptive study was conducted. All patients under 15 years old with foreign body ingestion (International Statistical Classification of Diseases and Related Health Problems; ICD-10 codes T18) treated in CMU Hospital from January 2006 to December 2017 were included. The data were analyzed using descriptive statistics. The guidelines were created, which paralleled the standard guidelines. *Results:* In total, 194 episodes of FB ingestion were recorded. These included 53.6% males and 46.4% females with a median age of 43.5 months. A history of foreign body ingestion complaints occurred in 77.8% of cases. Presentation was divided into asymptomatic (44.3%) and symptomatic (55.7%). The most common symptom was vomiting (23.2%). In the majority of cases, foreign bodies were located in the esophagus (37%). The most common type of foreign body was a coin (41.2%). Management included spontaneous passing (60.3%), endoscopy (35.6%), and others (3.1%). Complications before treatment were recorded in 9.3% of cases and after treatment in 2.1% of cases. *Conclusions:* Foreign body ingestion is common among children younger than four years old. Coins are the most common foreign body found, and the esophagus is the most common location. We recommend our created CMU Guidelines for management.

## 1. Introduction

Foreign body ingestion is one of the common problems among children. There is the greatest tendency for children between the ages six months and six years to have problems after placing objects in their mouths, this being the stage of exploratory development [1,2]. These events can cause serious complications [1,3].

Of the many kinds of objects found in such cases, which include coins, fish bones, pins, button batteries, magnets, household items, and many others, the most common objects found in most countries were coins [1,3,4,5]. However in some areas, batteries were commonly swallowed [6]. Ingested foreign bodies can lodge anywhere in the gastrointestinal (GI) tract, including the proximal esophagus, distal esophagus, and stomach. The diversity of the foreign bodies and lodging positions can cause different severities of complications [7]. This has led to a variation in management guidelines across many organizations [4,8,9,10,11].

Various presentations of patients with foreign body ingestion have been found. For example, vomiting, sensation of something being stuck, odynophagia, and dysphagia. Apart from having symptoms, some patients or their parents stated a history of ingestion.

History-taking and physical examination are the basic components of an initial assessment. Useful aspects of the history-taking include symptoms, type of foreign body, timing of presentation, and associated conditions. In the physical examination, patient status, vital signs, airway evaluation, signs of inability to manage their secretions, or emergency conditions, such as peritonitis or subcutaneous emphysema, are assessed [4,10]. In addition, plain radiography can be the most useful investigation. The radiograph demonstrates the location, number, size, and shape of any foreign bodies. This can help to exclude the presence of foreign bodies in airways and emergency conditions. One limitation is that some foreign bodies are not radiopaque subjects and cannot be seen from plain X-ray film. However, radiography is carried out in every single patient who is suspected to have ingested a foreign body. The view of the X-ray depends on the part of the body of concern. If location is not possible by X-ray, any radiolucent objects could be found using an esophagogram or computer tomography (CT) scan. Endoscopic removal can be carried out promptly in symptomatic cases and when the location of the foreign body is within endoscopic reach. Other investigations, such as ultrasonography and magnetic resonance imaging are unhelpful in this field [10].

Diversity in types of foreign body and organs in which foreign bodies lodge means there is variation in the management guidelines needed. The present published guidelines are widely practiced and useful, however, some controversial points exist within them. This study aimed to identify foreign body distribution in regards to type and location, together with the creation of the Chiang Mai University (CMU) Guidelines, improving the previous guidelines by combining with the newly gathered data.

## 2. Materials and Methods

This retrospective descriptive study was approved by the Research Ethics Committee of the Faculty of Medicine, CMU (Study Code: SUR-2561-05611/Research ID: SUR-2561-05611; date of approval: 14 August 2018). Our population included all pediatric patients under 15 years old with foreign body ingestion (International Statistical Classification of Diseases and Related Health Problems; ICD-10 codes T18) treated in CMU Hospital from January 2006 to December 2017. Patients attending the pediatric surgical unit, pediatric gastroenterology unit, and department of otolaryngology were included. Inpatient and outpatient department data were collected.

Characteristic data, associated conditions, presentation of patient, anatomic region, type of foreign body, management, complications, and outcome data were collected from the electronic database and reviewed. Patients with incomplete electronic data, toxic agent ingestion, emulsion ingestion, fish bones, history of ingestion but foreign body not identified, foreign body not in the GI tract, foreign body not ingested but other route, and patients who were more than 15 years old were excluded from the study.

Associated conditions included tracheoesophageal malformation, psychological problems, anatomical pathology in the GI tract, and previous abdominal surgery. Chief complaints were history of foreign body ingestion, sensation of something being stuck in the throat, dysphagia, vomiting, odynophagia, drooling, chest pain, and abdominal pain. Presentations of patients were divided into symptomatic and asymptomatic. The types of foreign body included coins, food bolus, button batteries, toys, marbles, magnets, safety pins, pins, hair pins, and cylindrical batteries. The types of foreign body were categorized as blunt, sharp, battery, magnet, and food bolus. Management strategies were spontaneous passage, endoscopic removal, and surgical removal. Outcomes were successful, unsuccessful, and unknown. Complications were identified before and after treatment.

Statistical analysis was performed using commercial statistical software (STATA 15.0; StataCorp LP, College Station, TX, USA). Categorical data were reported as count and percentage. Continuous data were reported as mean and standard deviation or median and interquartile range, according to data distribution.

Previous published guidelines were collected. The guidelines sourced were those from the North American Society for Pediatric Gastroenterology, Hepatology, and Nutrition (NASPGHAN) [8]; the European Society for Pediatric Gastroenterology, Hepatology, and Nutrition (ESPGHAN) [10]; the American Society for Gastrointestinal Endoscopy (ASGE) [4]; and the Wray Rural Training Tract Family Medicine Residency Program in Wray, CO, USA [11]. The accumulated data and these guidelines were reviewed and used to formulate the CMU Guidelines.

## 3. Results

**Figure 1:**
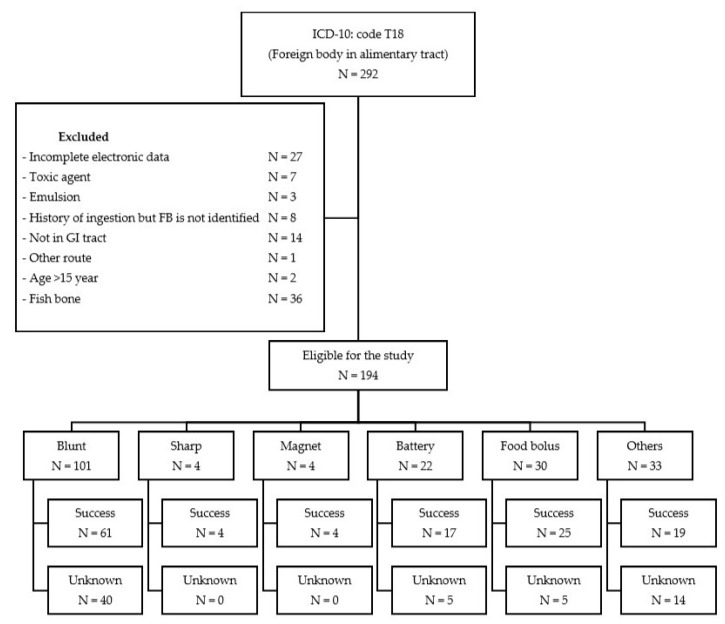
Study flow diagram showing distribution of ingested foreign bodies and result of management.

One hundred and ninety-four episodes of foreign body ingestion were recorded at CMU Hospital between January 2006 and December 2017, as shown in Figure 1. These were distributed into 104 (53.6%) males and 90 (46.4%) females. The median age was 43.5 months (range was 6 to 180 months). One hundred and twelve (57.7%) out of 194 episodes were in patients less than 48 months old. The peak age of ingestion was one to two years old, accounting for 21% of cases. The most frequent associated condition was tracheoesophageal malformation in 16 (8.3%) of 194 episodes. Most patients were from Chiang Mai Province, 138 episodes (71.1%). The characteristic data are summarized in Table 1.

Most patients were symptomatic (108 episodes, 55.7%). One hundred and fifty (77.8%) patients reported a history of foreign body ingestion. The second chief complaint was a sensation of something being stuck in the throat (8.8%). A summary of chief complaints is shown in Table 2. The most frequent symptom was vomiting (23.2%), followed by dysphagia, sensation of something being stuck in the throat, and cough. Time from ingestion to presentation was from five minutes to two weeks. The maximum time totaled two weeks, which included the time in a patient who was referred from another hospital.

Plain radiography, endoscopy, and laryngoscopy were performed to locate and manage foreign body ingestion. Table 3 shows the location of foreign bodies. Most objects were found in the esophagus (37%), stomach (29.2%), and jejuno-ileum (6.8%).

The most common type of foreign body ingested in our study was a coin (80 episodes, 41.2%), followed by food bolus (30 episodes, 15.5%), and a button battery (21 episodes, 10.8%) (Table 4). The distribution of types of foreign body is shown in Table 5. In this study, blunt objects were reported in the highest number of episodes (101 episodes, 52.1%), followed by food bolus (30 episodes, 15.5%).

The percentage of inpatient department patients comprised 58%, with outpatient department patients totaling 42%. In patients who were admitted, the median length of stay was one day (range from 1 to 16 days). Thirty percent (58 episodes) of patients were referred from another hospital. Forty-five percent of the referred cases were managed by spontaneous passage.

The primary management of overall foreign body ingestion was spontaneous passage, accounting for 117 episodes (60.3%). Reassurance and clinical treatment with fecal observations or follow-up radiography after a few days was advised. Endoscopic removal, including esophagoscopy, gastroscopy, and esophagogastroduodenoscopy, was performed in 69 episodes (35.6%). Surgical removal was performed in only two episodes (1.0%). The indication was bowel obstruction. The first case was caused by a tamarind seed and the second case by a cylindrical battery ingestion, which was lodged in the small bowel (Table 6).

All 134 episodes were successfully managed by various methods described in this study. Sixty (30.9%) episodes had unknown results due to being lost to follow-up (Table 6).

Complications before treatment included 18 (9.3%) episodes involving GI mucosal abrasions and bowel obstructions. Various degrees of mucosal injury of the oral, esophageal, and gastric mucosa ranged from redness, abrasion, ulceration, and necrosis. A coin (8/18 cases) located in the esophagus was the most common cause of pretreatment injury. Only one case of bowel obstruction was caused by food bolus. Four (2.1%) episodes with complications after treatment involved GI mucosal abrasions and aspiration pneumonia, and three cases of esophageal and gastric mucosal abrasion were found. One case of aspiration pneumonia occurred in a case involving a coin located in the esophagus (Table 6).

**Figure 2:**
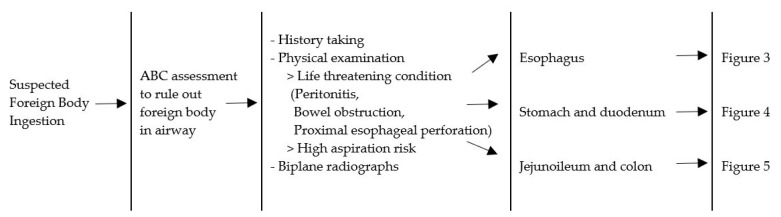
Management algorithm for foreign body ingestion. A = airway; B = breathing; C = circulation.

**Figure 3:**
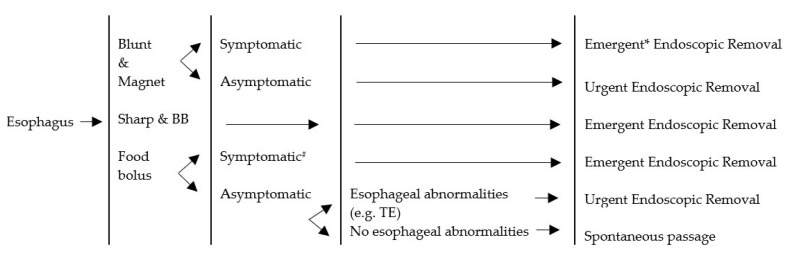
Management algorithm for foreign bodies in esophagus. BB = button battery; TE = tracheoesophageal malformation; # Symptomatic = cannot manage secretion, sore throat; * Emergent = within 2 h; Urgent = within 24 h; Elective = more than 24 h.

**Figure 4:**
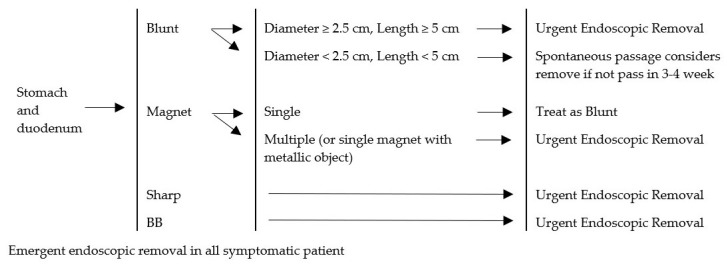
Management algorithm for foreign bodies in stomach and duodenum. BB = button battery.

**Figure 5:**
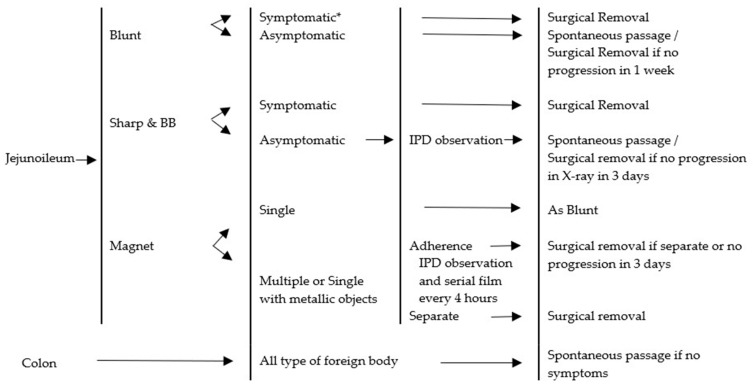
Management algorithm for foreign bodies beyond the duodenum. BB = button battery. IPD = inpatient department. * Symptomatic; peritonitis, bowel obstruction.

Four guidelines were reviewed. The comparative tables of four guidelines and the proposed CMU Guidelines are shown in Table 7. With regard to the esophagus, a similarity among the guidelines was found. It was suggested in all cases that esophageal foreign bodies were removed. The difference was only the timing of the intervention. In our study, esophageal foreign bodies caused esophageal ulceration when the contact time neared 24 h. We recommended the removal of esophageal foreign bodies before 24 h. Concerning food bolus, in the case of asymptomatic impaction, a normal esophagus and dissolvable food treatment would usually be by spontaneous passage. In this study, five cases of esophageal food impaction were passed spontaneously. The objects involved were mostly candy and the patients presented with no symptoms at the time. Regarding the stomach and duodenum, we recommended removing any object longer than 5 cm. The previous guidelines recommended removing 6 cm long objects because this length could not pass the duodenal sweep. We found one eight-year-old child with a 5 cm hair pin stuck in the duodenal sweep, a case subsequently treated by endoscopic removal. In cases concerning a battery located in the stomach, we found two that involved antral ulcers, even though contact time was 5 and 6 h, respectively. We recommended removing the battery in the stomach to prevent any chemical injury from the uncertain quality of the button battery. The CMU Guidelines are described in Figure 2, Figure 3, Figure 4 and Figure 5.

## 4. Discussion

Foreign body ingestion can be found at any age, but is more frequent among children aged from six months to four years old [1,8,9]. In our study, 194 episodes had occurred over 12 years (January 2006 to December 2017). The peak prevalence was from 12 months old to 24 months old. The data were collected from the pediatric surgical unit, pediatric gastroenterology unit, and department of otolaryngology. Treatment algorithms were similar among divisions.

In related studies, patients involving foreign body ingestion presented with vomiting, drooling, dysphagia, cough, abdominal pain, sensation of something being stuck in the throat, hematemesis, and history of foreign body ingestion [1,2,3,11]. In our study, the most frequent chief complaint was having a history of foreign body ingestion (77.84%). According to the ESPGHAN Guidelines, vomiting and drooling are the predominant symptoms [10]. One hundred and eight (55.67%) of all patients presented with symptoms. The most common symptom was vomiting (45, 23.20%), as was also noted in the ESPGHAN Guidelines.

In 2011, the ASGE Standards of Practice Committee as well as the guidelines from the Royal Children’s Hospital Melbourne suggested that patients having anatomical anomalies, such as tracheoesophageal malformation, had a high risk of having objects stuck in their gastrointestinal (GI) tract [4,9]. In our study, patients who had tracheoesophageal malformations had multiple episodes—up to four episodes in this dataset.

We divided the foreign bodies into five groups: blunt, sharp, magnet, battery, and food bolus. This categorization is similar to the ESPGHAN, NASPGHAN, and ASGE reports [4,8,10]. The ESPGHAN Guidelines have one more category, which is drug packets [10]. The NASPGHAN Guidelines also have one additional category, namely, a superabsorbent object [8]. The ASGE Guidelines incorporate two more categories, namely, drug packets and coins [4]. In our study, no incidence of drug packet ingestion was found, and two episodes of absorbable balls were observed. These numbers were too small to categorize and were placed in the “others” group. The blunt object category comprised coins, hair pins, marbles, and toys. Safety pins and pins were included in the sharp object category, however, a closed safety pin was considered to be blunt object. Single and multiple magnets were observed. Batteries were divided in two categories, button battery and cylindrical battery. The management algorithm for foreign body ingestion was categorized by organ, involving the esophagus, stomach, duodenum, and beyond the stomach and duodenum. The initial evaluation is described in Figure 2.

Coins (41.24%) were the most common foreign bodies ingested, followed by food bolus (15.46%) and button batteries (10.82%), findings similar to many studies [1,2,3,8,10]. On the other hand, button battery ingestion was the most common foreign body in some countries where bank notes were used instead of coins [6]. The use of coin currency is common in Thailand and was, therefore, relevant to our results.

In many studies, the most common region where a foreign body found was the esophagus [1,2,3,5,6]. Again this was comparable with our study, in which the esophagus (36.98%) was also the most frequent location for an ingested foreign body. The second most common was the stomach (29.17%). The most frequent foreign body lodged in the esophagus was a coin. The average time from ingestion to presentation in the esophagus was four hours. The minimum time was five minutes and the maximum was seven days, which included referred cases. All esophageal foreign bodies involved attempted removal. Fifty-six (78.87%) out of the 71 foreign bodies located in the esophagus were removed by endoscopic removal. Others were removed by direct laryngoscopy and spontaneous passage before intervention. In this study, all foreign bodies in the esophagus involved blunt objects and food boluses. As described in the ESPGHAN Guidelines, a foreign body involving a blunt object was removed by endoscopic removal in every case [10]. The NASPGHAN Guidelines recommend urgent removal of blunt objects by endoscopic removal when patients present with symptoms [4,8]. When the patient had no symptoms, the management was observation for 12–24 h. If the foreign body was still in the esophagus after 24 h, it would be removed endoscopically to prevent complications [4,8]. We recommend that blunt objects in the esophagus should be removed due to the possibility of complications. One of our cases was complicated by aspiration pneumonia, requiring treatment and extension of the length of stay. Time of removal was considered as an emergency in symptomatic cases and within 24 h in case of asymptomatic patients. When the foreign body is a food bolus, we also recommend removal. In our study, patients with tracheoesophageal malformation frequently had food particle obstructions. However, patients who were symptomatic with a normal esophagus should also be treated by endoscopic removal to prevent secretion aspiration. Time of removal was the same as that recommended for coins. Almost all foreign bodies in the esophagus were removed endoscopically. Most patients arrived with symptoms or silent symptoms that could not be identified. There was a strong possibility of complications, such as esophageal injury, erosion, and perforation when the foreign body remained in the esophagus for 24 h [8,10]. The foreign bodies in the lower esophagus were more likely to pass by themselves [4]. In our study, a coin in the lower esophagus passed spontaneously before 24 h.

In our series, presence of a foreign body in the stomach and duodenum occurred in 62 episodes. Thirty-eight episodes involved blunt objects. Thirty-two episodes out of the 38 passed spontaneously, while four episodes were removed endoscopically. All blunt object sizes were under 2.5 cm in diameter and could pass without assistance in 32 episodes of this report [4,8,10]. Endoscopic removal was performed in four episodes and occurred for a 5-cm-long hair pin [11]. Three coins were removed due to parental concerns. Three sharp objects were ingested and located in the stomach. Two episodes passed spontaneously involving closed safety pins. These were treated as blunt objects because the sharp edge was inside its case. Another one involved a metallic pin removed by endoscopy. Two magnets were ingested, one of which was a single magnet with spontaneous treatment. The other episode involved multiple magnets with endoscopic removal. Multiple magnets with separation could lead to bowel wall necrosis with fistula formation, perforation, obstruction, volvulus, or peritonitis, Ikenberry and Thomson et al. [4,10] requiring removal. Fourteen episodes of button battery ingestion occurred with nine of the 14 passing spontaneously within 48 h. Others were performed by endoscopic removal. According to many guidelines, when the button battery size is more than 2 cm in diameter, the patient should be observed for 48 h. After 48 h if the battery remained in the stomach, then endoscopic removal would be performed [4,8,10]. Other types of foreign bodies passed spontaneously, and endoscopic removal depended on the decision of physician (Figure 4). Hazardous foreign bodies in the stomach and duodenum, which an endoscope could reach, were managed endoscopically. Three guidelines [4,8,10] regarding foreign bodies in the stomach and duodenum mentioned the size of the object. Any object larger than 2.5 cm in diameter or 6 cm long were removed in every case. On the other hand, the Colorado guidelines defined the need for removal of a long object by a sliding scale of length against age. For a child younger than one year old, an object needing removal is larger than 2 cm in diameter or longer than 3 cm. From one year old and older, the guideline is between 3 and 5 cm. This guideline is also recommended to remove all long objects before the duodenum [11]. We recommend removing a foreign body wider than 2.5 cm and longer than 5 cm in all age groups. The reasoning behind this is that the large object may not pass the pyloric canal and the long object may become stuck in the duodenal sweep, as in the case with a 5-cm-long hairpin in our series. Endoscopic removal of button batteries is recommended in every case due to the uncertainty of the quality of battery. Many guidelines recommend observation by repeated films, with timing to repeat a film dependent on the size and age of the child. We recommend treatment of a cylindrical battery be applied to a button battery. A single magnet is recommended to be treated as a blunt object.

Most foreign bodies beyond the stomach and duodenum, including the jejunum, ileum, and colon, passed spontaneously in our study. However, one episode involved an attempted removal by endoscopy because the first film showed a button battery in the antrum of the stomach. Two episodes involving button batteries required surgical treatment due to bowel obstruction. For patients who have ingested a hazardous foreign body, such as a cylindrical battery, close observation of any abdominal signs is recommended. When any abdominal signs are presented, there is a recommendation for surgery (Figure 5). ASGE Guidelines recommend removing blunt objects remaining in the same location of the small bowel for more than one week, even when asymptomatic [4]. NASPGHAN Guidelines recommend observation [8]. In our study, we have followed the NASPGHAN Guidelines.

Apart from the organs mentioned above, two episodes of sunflower seed shell and a wooden piece were identified in the tonsils. They were seen by physical examination and removed by forceps.

This study employed a retrospective descriptive design. The limitations were missing data during data collection and unknown results of spontaneous treatment because they were lost to follow-up. We could not establish guidelines for radiolucent and superabsorbent objects due to there being too few cases to formulate a conclusion.

## 5. Conclusions

In conclusion, foreign body ingestion is common among children younger than four years old. According to our results, coins were the most common foreign body found, and the esophagus was the most frequent location. We recommended foreign object management according to our created guidelines.

## Figures and Tables

**Table 1:** Personal data.

Characteristics	Count (*N* = 194)	Percentage (%)
**Sex**		
Male	104	53.61
Female	90	46.39
**Age range (months)**		
Median (IQR *)	43.5 (21–72)	
≤48 months	112	57.73
>48 months	82	42.27
**Associated condition**		
Tracheoesophageal malformation	16	8.25
Psychotic problem	3	1.55
Anatomical pathology in the GI tract	2	1.03
Previous abdominal surgery	1	0.52
**Present address**		
Chiang Mai	138	71.13
Northern provinces ** (except Chiang Mai)	45	23.20
Others	11	5.67
**Type of visit**		
Walk-in	136	70.10
Referred	58	29.90

* IQR = interquartile range. ** Northern provinces: Chiang Rai, Lampang, Lamphun, Mae Hong Son, Nan, Phayao, Phrae.

**Table 2:** Chief complaint and presentation.

	Count (*N* = 194)	Percentage (%)
**Chief complaint**		
History of foreign body ingestion	150	77.84
Sensation of something being stuck in the throat	17	8.76
Dysphagia	10	5.15
Vomiting	10	5.15
Odynophagia	2	1.03
Drooling	1	0.52
Chest pain	1	0.52
Abdominal pain	1	0.52
Other	1	0.52
**Presentation**		
**Symptomatic**	108	55.67
Vomiting	45	23.20
Dysphagia	27	13.92
Sensation of something being stuck in the throat	26	13.40
Cough	13	6.70
Drooling	12	6.19
Odynophagia	9	4.64
Abdominal pain	8	4.12
Chest pain	6	3.09
Other	29	14.95
**Asymptomatic**	86	44.33

**Table 3:** Location of foreign body.

Location of Foreign Body	Count (*N* = 192)	Percentage (%)
Esophagus	71	36.98
Stomach	56	29.17
Jejuno-ileum	13	6.77
Colon	9	4.69
Duodenum	6	3.13
Tonsil	2	1.04
Other	35	18.23

Note: total = 192 cases, unknown location = 2 cases due to asymptomatic radiolucent blunt objects (plastic coin and candy).

**Table 4:** Type of foreign body.

Type of Foreign Body	Count (*N* = 194)	Percentage (%)
Coin	80	41.24
Food bolus	30	15.46
Button battery	21	10.82
Toys	12	6.19
Marble	8	4.12
Magnet	4	2.06
Safety pin	3	1.55
Pin	1	0.52
Hair pin	1	0.52
Cylindrical battery	1	0.52
Other *	33	17.01

* Other: chewing gum, dental bur, LED light, metallic nail, pencil lead, plastic piece, fruit seed, round button, rubber band, screw, staple pin, seed coat, wire ring, wooden piece, paper, clay, metallic piece of necklace, absorbable ball, silver ring.

**Table 5:** Groupings of foreign bodies according to management.

Type of Foreign Body	Count (*N* = 194)	Percentage (%)
Blunt	101	52.06
Food bolus	30	15.46
Battery	22	11.34
Sharp	4	2.06
Magnet	4	2.06
Other *	33	17.01

* Other: chewing gum, dental bur, LED light, metallic nail, pencil lead, plastic piece, fruit seed, round button, rubber band, screw, staple pin, seed coat, wire ring, wooden piece, paper, clay, metallic piece of necklace, absorbable ball, silver ring.

**Table 6:** Management, outcome, and complication.

	Count (*N* = 194)	Percentage (%)
**Management**		
Spontaneous	117	60.31
Endoscopic removal	69	35.57
Surgical removal	2	1.03
Other *	6	3.09
**Outcome**		
Successful	134	69.07
Unknown	60	30.93
Unsuccessful	0	0
**Complication**		
Before treatment	18	9.28
After treatment	4	2.06

* Other: forceps removal, direct laryngoscopy.

**Table 7:** (a) Comparison between guidelines: esophagus. (b) Comparison between guidelines: stomach and duodenum. (c) Comparison between guidelines: beyond duodenum.

**(a)**						
	**Guideline**	**NASPGHAN [8]**	**ESPGHAN [10]**	**ASGE [4]**	**Colorado [11]**	**CMU**
**Type/Location**						
**Esophagus**						
Blunt						
Symptomatic		Urgent Endoscopic Removal	Emergent Endoscopic Removal	Emergent Endoscopic Removal	All in esophagus should be removed (endoscopic/Foley’s catheter) or push in stomach (bougienage) within 24 h	Emergent Endoscopic Removal
Asymptomatic		Within 24 h Endoscopic Removal	Urgent Endoscopic Removal	Urgent Endoscopic Removal or Push in Stomach		Urgent Endoscopic Removal
Button battery						
Symptomatic and Asymptomatic		Emergent Endoscopic Removal	Emergent Endoscopic Removal	Emergent Endoscopic Removal		Emergent Endoscopic Removal
Magnet		Management in esophagus and stomach was in the same category				
Symptomatic		See Stomach	As blunt in single, Urgent Removal all endoscopic reach for multiple magnets	Urgent Removal all endoscopic reach		Emergent Endoscopic Removal
Asymptomatic						Urgent Endoscopic Removal
Sharp						
Symptomatic and Asymptomatic		Urgent Endoscopic Removal	Emergent Endoscopic Removal	Emergent Endoscopic Removal		Emergent Endoscopic Removal
Food Bolus						
Symptomatic		Urgent Endoscopic Removal and workup for esophageal abnormality e.g., water soluble contrast, esophageal biopsy	Emergent Endoscopic Removal	Endoscopic Removal or Push in Stomach or Glucagon		Emergent Endoscopic Removal
Asymptomatic			Urgent Endoscopic Removal			Esophageal abnormalities: Urgent Endoscopic Removal No esophageal abnormalities: spontaneous passage
**(b)**						
	**Guideline**	**NASPGHAN [8]**	**ESPGHAN [10]**	**ASGE [4]**	**Colorado [11]**	**CMU**
**Type/Location**						
**Stomach and Duodenum**						
Blunt						
Symptomatic		Spontaneous Passage Endoscopic Removal if - diameter > 2.5 cm, longer than 6 cm - does not pass in 2–4 weeks	Endoscopic Removal	Endoscopic Removal if - diameter > 2.5 cm, longer than 6 cm - does not pass in 3–4 weeks	Endoscopic Removal	Urgent Endoscopic Removal
Asymptomatic			Spontaneous Passage Endoscopic Removal if - diameter > 2.5 cm, longer than 6 cm - does not pass in 4 weeks		Endoscopic Removal in large object: - Age < 1 year old; 2–3 cm - Age ≥ 1 year old; 3–5 cm	Spontaneous Passage Urgent Endoscopic Removal if - diameter ≥ 2.5 cm, longer than 5 cm - not pass in 3–4 weeks
Batteries						
Symptomatic		Age < 5 years old and BB ≥ 20 mm—Urgent Endoscopic Removal Age ≥ 5 years old and BB < 20 mm—Observation repeat film at 48 h, remove if persists or patient presents symptoms	Emergent Endoscopic Removal	Endoscopic Removal	Endoscopic Removal	Urgent Endoscopic Removal
Asymptomatic			- Known GI abnormalities - Simultaneously swallowed with the magnet - BB > 20 mm repeat film at 48 h, remove if persists - CB—repeat film at 7–14 days	Observation repeat film at 48 h, Endoscopic Removal if persists	Endoscopic Removal if no progression in 3–4 days	Urgent Endoscopic Removal
Magnet						
Single		Spontaneous Passage or Removal if risk for further ingestion	Spontaneous Passage	Urgent Endoscopic Removal	-	As blunt
Multiple		Endoscopic Removal	Endoscopic Removal			Urgent Endoscopic Removal
Sharp						
Symptomatic and Asymptomatic		Endoscopic removal (unless short weighted blunt one end)	Emergent Endoscopic Removal	Urgent Endoscopic Removal	Endoscopic Removal	Urgent Endoscopic Removal
**(c)**						
	**Guideline**	**NASPGHAN [8]**	**ESPGHAN [10]**	**ASGE [4]**	**Colorado [11]**	**CMU**
**Type/Location**						
**Beyond Duodenum**						
Blunt						
Symptomatic		Surgical Removal (or enteroscopy)	-	Surgical Removal if located in the same location longer than 1 week	Surgical Removal if large object with weekly repeat film and no progression more than 1 week	Surgical Removal
Asymptomatic		Spontaneous Passage	-			Spontaneous Passage/Surgical Removal if no progression in 1 week
Batteries						
Symptomatic		Removal	-	-	Removal	Surgical Removal
Asymptomatic		Observation repeat film at 48 h if persist consider removal	-	-	Removal if repeat film and no progression in 3–4 days	Spontaneous passage in IPD Surgical removal if no progression in X-ray in 3 days
Magnet						
Single		Spontaneous Passage	Spontaneous Passage and Surgical Consultation	-	-	As blunt
Multiple		Symptomatic—refer to pediatric surgeon Asymptomatic—enteroscopy/repeat film every 4–6 h, remove if no progression				Adherence—IPD observation, repeat film every 4 h, remove if no progression in 3 days or separate Separate—Surgical Removal
Sharp						
Symptomatic		Enteroscopy/Surgical Removal	Enteroscopy/Surgical Removal	Enteroscopy/Surgical Removal	Removal	Surgical Removal
Asymptomatic		Spontaneous Passage, remove if no progression in 3 days	Spontaneous Passage in IPD setting, remove if no progression in 3 days	Spontaneous Passage, remove if no progression in 3 days	Spontaneous Passage, daily radiograph remove if no progression in 3 days	Spontaneous Passage in IPD Surgical removal if no progression in X-ray in 3 days

GI—gastrointestinal; BB—button battery; CB—cylindrical battery; IPD—inpatient department.
